# Complete plastome sequence of *Wrightia laevis* Hook. f. a dyestuff species

**DOI:** 10.1080/23802359.2020.1778578

**Published:** 2020-06-22

**Authors:** Lin-Ming Li, Jie-Xiong Fu, Xi-Qiang Song

**Affiliations:** a School of Life and Pharmaceutical Sciences, Hainan University, Haikou, China; b Hainan Provincial Forestry Project Management Office, Haikou, China; c Key Laboratory of Genetics and Germplasm Innovation of Tropical Special Forest Trees and Ornamental Plants (Ministry of Education), College of Forestry, Hainan University, Haikou, China

**Keywords:** *Wrightia laevis*, plastome, phylogeny, genome structure, apocynaceae

## Abstract

*Wrightia laevis* Hook. f. is a great tree of Apocynaceae. It is mainly distributed in Southeast provinces of China and Southeast Asian countries. It is a plant that combines dyestuff and economic value. There is no study on the genome of *W. laevis*so far. Here we report and characterize the complete plastid genome sequence of *W. laevis* in order to provide genomic resources useful for promoting its conservation. The complete chloroplast genome of *W. laevis* is 155,274 bp in length with a typical quadripartite structure, consisting of a large single-copy region (LSC, 85,463 bp), a single-copy region (SSC, 18,181 bp) and a pair of inverted repeats (IRs, 25,815 bp). There are 133 genes annotated, including 88 unique protein-coding genes, 8 unique ribosomal RNA genes, and 37 transfer RNA genes. The overall G/C content in the plastome of *W. laevis* is 38.05%. The complete plastome sequence of *W. laevis* will provide a useful resource for the conservation genetics of this species as well as for phylogenetic studies in Apocynaceae.

*Wrightia laevis* Hook. f. is a plant of the family Apocynaceae, mainly distributed in Southeast provinces of China and Southeast Asian countries. It is a plant that combines medicinal and dyestuff value (Li et al. 1995). The chloroplast genome sequence carries rich information for plant molecular systematics and Barcoding. There have been no studies on the genome of *W. laevis* up to now. To provide a rich genetic information and improve *W. laevis* molecular breeding in the future, we report and characterize the complete plastid genome sequence of *W. Laevis* (GenBank accession number: MT505711).

In this study, the fresh leaves of *W. laevis* were collected from Jianfeng mountain in Hainan province (108.89° E, 18.73° N). A voucher specimens (HUTB 187223) were deposited in the Herbarium of the Institute of Tropical Agriculture and Forestry (code of herbarium: HUTB), Hainan University, Haikou, China.

The experiment procedure was as reported in Wang et al. (2019). Total DNA of the *W. laevis* was sequenced with second-generation sequencing technology (Illumina HiSeq 2000, San Diego, CA). The chloroplast genome sequence reads were assembled with bioinformatic pipeline including SOAP2 software (Li et al. 2009) and several runs of manual corrections of sequence reads. Genes encoded by this genome were annotated by import the fasta format sequence to the DOGMA (Wyman et al. 2004) and recorrected by manual. The results showed that plastome of *W. laevis* possess a total length 155,274 bp with the typical quadripartite structure of angiosperms, containing two inverted repeats (IRs) of 25,815 bp, a large single copy (LSC) region of 85,463 bp and a small single copy (SSC) region of 18,181 bp. The plastome contains 133 genes, consisting of 88 unique protein-coding genes, 37 unique tRNA genes and 8 unique rRNA genes. The overall G/C content in the plastome of *W. laevis* is 38.05%, which the corresponding value of the LSC, SSC, and IR region were 36.20, 31.80, and 43.40%, respectively.

We used RAxML (Stamatakis 2006) with 1000 bootstraps under the GTRGAMMAI substitution model to reconstruct a maximum likelihood (ML) phylogeny of 12 published complete plastomes of Apocynaceae, using *Gentianaapiata* (Gentianaceae) as outgroups. According to the phylogenetic topologies, *W. laevis* was closely related to *W. natalensis*. Most nodes in the plastome ML trees were strongly supported (Figure 1). The complete plastome sequence of *W. laevis* will provides a useful resource for the conservation genetics of this species as well as for the phylogenetic studies for Apocynaceae.

**Figure 1.**
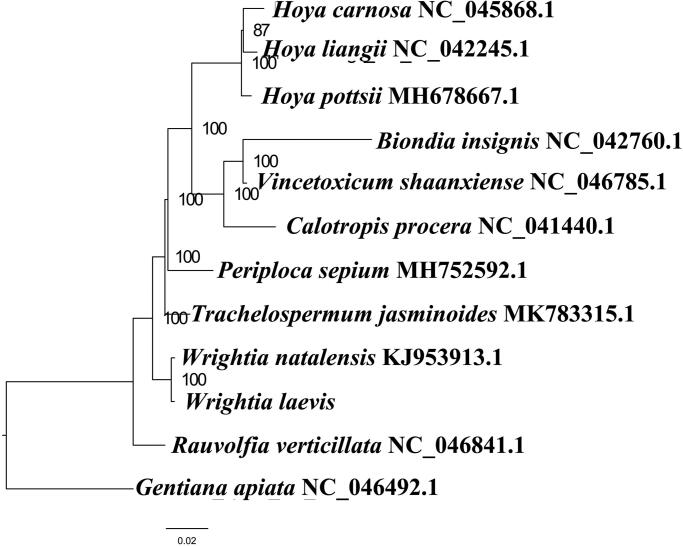
Maximum likelihood phylogenetic tree based on 12 complete chloroplast genomes. Accession number: *Wrightia laevis* (this study); *Biondia insignis* NC_042760.1; *Calotropis procera* NC_041440.1; *Hoya carnosa* NC_045868.1; *Hoya liangii* NC_042245.1; *Hoya pottsii* MH678667.1; *Periploca sepium* MH752592.1; *Rauvolfia verticillata* NC_046841.1; *Trachelospermum jasminoides* MK783315.1; *Vincetoxicum shaanxiense* NC_046785.1; *Wrightia natalensis* KJ953913.1; outgroup: *Gentiana apiata* NC_046492.1.The number on each node indicates the bootstrap value.

## Data Availability

The data that support the findings of this study are openly available in GenBank of NCBI at http://www.ncbi.nlm.nih.gov, reference number MT505711.
